# Draft Genome Sequence of the New Pathogen for Bivalve Larvae *Vibrio bivalvicida*

**DOI:** 10.1128/genomeA.00216-16

**Published:** 2016-04-07

**Authors:** Javier Dubert, Edward J. Spinard, David R. Nelson, Marta Gomez-Chiarri, Jesús L. Romalde, Juan L. Barja

**Affiliations:** a Departamento de Microbiología y Parasitología, CIBUS–Facultad de Biología, Universidade de Santiago de Compostela, Santiago de Compostela, Spain; b Department of Cell and Molecular Biology, University of Rhode Island, Kingston, Rhode Island, USA; c Department of Fisheries, Animal and Veterinary Sciences, University of Rhode Island, Kingston, Rhode Island, USA

## Abstract

*Vibrio bivalvicida* is a novel pathogen of bivalve larvae responsible for recent vibriosis outbreaks affecting shellfish hatcheries. Here, we announce the draft genome sequence of *V. bivalvicida* 605^T^ and describe potential virulence factors.

## GENOME ANNOUNCEMENT

The genus *Vibrio* is the largest member of the family *Vibrionaceae* and comprises more than 126 bacterial species and 2 subspecies (http://www.bacterio.net/vibrio.html) clustered in 18 clades and 4 orphan species (1, 2). Vibrios are widespread in marine environments, showing high diversity at the metabolic and ecological levels, and their association with marine bivalves has regularly been reported (3). Vibriosis caused by some *Vibrio* spp. represents the main bottleneck of the production process at bivalve hatcheries due to the rapid colonization of the bivalve larvae by these pathogens, leading to high larval mortality rates and the loss of production batches (4). The Orientalis clade has a relevant significance for bivalve aquaculture since it includes some of the most well-known larval pathogens, such as *Vibrio tubiashii* subsp. *tubiashii*, *V. tubiashii* subsp. *europaeus*, and the recently described *V. bivalvicida*, whose broad range of action was reported in larvae of different bivalve species (5).

*V. bivalvicida* 605^T^ (= CECT 8855^T^ = CAIM 1904^T^) was originally isolated from an episode of larval mortality of carpet shell clam (*Ruditapes decussatus*) in a bivalve hatchery located in Galicia (northwest Spain). Genomic DNA was sequenced using an Illumina MiSeq sequencer by Sistemas Genómicos (Valencia, Spain) with 100× coverage. Reads were trimmed for quality, ambiguous nucleotides, and adapters and were assembled using SPAdes version 3.6 (6). QUAST (7) was used to evaluate the assembly. The assembly produced 91 contigs totaling 4,922,047 bp with an average G+C content of 43.6%. The *N*_50_ contig size is 191,755 bp, with the largest contig being 691,504 bp. The resulting draft genome sequence was annotated with the Rapid Annotations using Subsystems Technology (RAST) server, resulting in 4,619 open reading frames (8).

The genome of *V. bivalvicida* 605^T^ encodes three putative extracellular proteins that have been characterized in other *Vibrio* spp.: a phospholipase/hemolysin that shows similarity to Plp in *V. anguillarum* M93Sm (9), a hemolysin annotated as HlyA that shows similarity to Vah1 in *V. anguillarum* M93Sm (9), and a metalloprotease that shows similarity to VtpA in *V. coralliilyticus* RE22 (10). Additional putative extracellular virulence factors identified by RAST are five hemolysins and two phospholipases. Two secretion systems (type III and type VI) used to deliver effectors directly into a host cell were identified on the *V. bivalvicida* 605^T^ genome. The only conserved domain annotated in the type III–injected virulence protein is the YopH N-terminal domain (11). This domain is needed for translocation from the bacterium into the eukaryotic host cell.

This work constituted the first draft genome description of the novel larval pathogen of bivalves *V. bivalvicida* 605^T^. This genome information contributes to the study of virulence factors, the development of new accurate diagnostic methods, and the knowledge of the bivalve pathogenic *Vibrio* spp.

### Nucleotide sequence accession numbers.

This whole-genome shotgun project has been deposited in DDBJ/EMBL/GenBank under the accession number LLEI00000000. The version described in this paper is the second version, LLEI02000000.
